# Synergistically Optimizing the Thermoelectric Performance of n‑Type SnS through an Integrated Systematic Approach

**DOI:** 10.1021/acsami.5c21755

**Published:** 2026-02-18

**Authors:** Sidharth Duraisamy, Yang-Yuan Chen, Kuei-Hsien Chen, Maw-Kuen Wu, G. Peramaiyan, V. K. Ranganayakulu, Muluken Biadgelegn Wollele, Min-Nan Ou

**Affiliations:** † Institute of Physics, 38017 Academia Sinica, 11529 Taipei, Taiwan; ‡ Institute of Atomic and Molecular Sciences, Academia Sinica, 10617 Taipei, Taiwan

**Keywords:** n-type SnS, sulfur vacancies, halogen doping, selenium alloying, synergistic combination, thermoelectric materials

## Abstract

Tin­(II) sulfide (SnS) is a promising p-type semiconductor known for its high thermoelectric performance and eco-friendly properties, offering a viable alternative to group IV–VI compounds. However, achieving n-type conductivity in SnS has been challenging due to the propensity for native Sn vacancies. This study addresses this challenge by synthesizing polycrystalline n-type SnS_1−δ_ (δ = 0.05 and 0.075) samples through solid-state reaction. By introducing sulfur vacancies to counteract Sn vacancies, followed by aliovalent (Cl^–^) and isoelectronic (Se^2–^) substitutions, we significantly enhance the thermoelectric performance of n-type SnS. Chlorine doping further improves electrical conductivity, with SnS_0.455_Se_0.45_Cl_0.02_ showing superior performance. Additionally, incorporating 0.03 mol % SnCl_2_ in SnS_0.475_Se_0.45_ compensates for intrinsic Sn vacancies, optimizing the power factor and lowering lattice thermal conductivity. Consequently, we realized a figure of merit *ZT* (*ZT*_max_) of ≈0.7 at 823 K and an average *ZT* (*ZT*_ave_) of ≈0.2 from 308 to 823 K, the highest reported values for n-type SnS. This work advances the optimization of n-type SnS and lays the groundwork for developing SnS-based thermoelectric devices.

## Graphical abstract

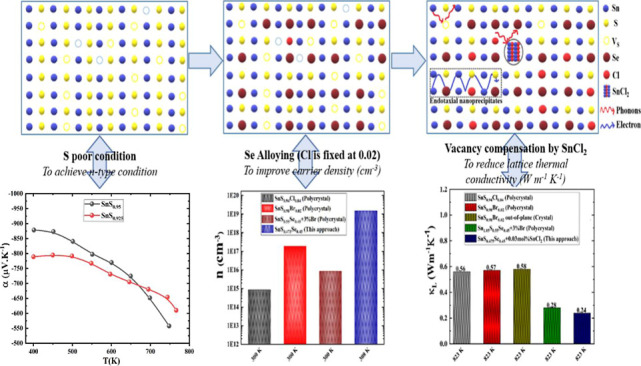

## 1 Introduction

Thermoelectric technology exemplifies an advanced paradigm in sustainable power generation, wherein charge carriers serve as working fluids to facilitate the direct transduction of thermal energy into electrical output, thereby offering a potential solution to energy and environmental challenges. The optimal materials for thermoelectric generators are characterized by a high dimensionless figure of merit *ZT*, expressed as *ZT* = (*S* ^2^σ*T*/*K*), wherein *S* denotes the Seebeck coefficient, σ represents the electrical conductivity, *K*_e_ signifies the electronic thermal conductivity, *K*_l_ corresponds to the lattice thermal conductivity, and *T* designates the absolute temperature in the system. Since the efficiency of thermoelectric materials depends on the interplay of electrical and thermal transport carriers in both p- and n-type materials, the thermal carriers, particularly phonons, often impose a primary constraint on conversion efficiency.

Recent decades have witnessed remarkable advances in thermoelectric materials synthesis, with *ZT* values reaching or exceeding 2 through innovative design and advanced fabrication techniques.^1^ Although several high-performance Group IV–VI compounds such as lead Chalcogenide,^2–9^ GeTe,^10,11^ and SnTe^12–14^ have been widely studied, their large scale application is limited by the toxicity of Pb and the scarcity and high cost of Ge and Te. In this context, SnSe has attracted significant attention from thermoelectric community, as it exhibits high *ZT* value in both p and n-type forms owing to its strong lattice anharmonicity,^15–17^ multiband electronic transport,^18–20^ and unique three-dimensional charge and two-dimensional phonon transport.^21–23^ As a sulfur analogue of SnSe, SnS shares a similar crystal and electronic structure while offering additional advantages of low cost, high earth abundance, and better environmental compatibility, making it an attractive candidate for large scale thermoelectric application.^23^ In particular, p-type SnS has recently demonstrated outstanding thermoelectric performance, achieving a *ZT* of approximately 1.6 at 873 K and an average *ZT* of 1.25 over the temperature range of 300–873 K in SnS_0.91_Se_0.09_ alloys.^23^ These exceptional results highlight the strong potential of SnS as a sustainable thermoelectric material system.

Despite extensive efforts to enhance the electrical transport performance of p-type SnS, effective n-type conversion remains challenging due to the inherently low formation energy of Sn vacancies.^24–26^ Under sulfur-rich conditions, Sn vacancies exhibit lower formation energies compared to sulfur vacancies, acting as shallow acceptors.^24–26^ Conversely, under sulfur-poor conditions, sulfur vacancies, although having lower formation energies, fail to compensate for hole carriers generated by Sn vacancies due to their states being closer to the valence band maximum.^24–26^ To enhance n-type conductivity, strategic modification of the defect chemical environment within SnS is required. This includes doping with transition metals and halogens (Cl and Br), as well as optimizing the sintering temperature. However, despite these efforts, halogen-doped SnS systems such as SnS_0.96_Cl_0.04_ and SnS_0.98_Br_0.02_ still exhibit thermally activated transport and limited carrier mobility, resulting in suboptimal Z*T*_max_ values of only ∼0.2 at 823 K.^27,28^ Although n-type Bi_2_Te_3_ remains the benchmark thermoelectric material at near-room temperature, its performance rapidly degrades at elevated temperatures and it relies on tellurium, a scarce and expensive element. Consequently, Bi_2_Te_3_ cannot serve as a sustainable or temperature-matched counterpart for high-temperature p-type SnS. For optimal thermoelectric device performance and long-term stability, fabricating both p-type and n-type legs from the same material system is highly advantageous, as it minimizes thermal expansion mismatch, interfacial degradation, and chemical incompatibility. In this context, developing high-performance n-type SnS is not merely desirable but essential for realizing fully SnS-based, earth-abundant, and environmentally benign thermoelectric modules operating in the midto-high temperature range. Although SnS and SnSe share the same crystal and electronic framework, SnSe exhibits much stronger lattice anharmonicity,^20,29–31^ more favorable band convergence,^32–35^ and weaker self-compensation,^25^ resulting in superior thermoelectric performance. Se alloying enables SnS to progressively inherit these SnSe like transport characteristics while retaining the sulfur-rich host lattice.^36,37^

In this study, we successfully synthesized n-type SnS_1−δ_ polycrystalline materials with enhanced anisotropic properties through a three-step process. First, we achieved a controlled transition to n-type conductivity by precisely adjusting the sulfur vacancy concentration (δ). Second, we employed dual substitution (aliovalent and isoelectronic alloying) on SnS_0.925_. Finally, we added metal chloride (SnCl_2_) for dual vacancy compensation. We systematically synthesized and characterized a series of sulfur-deficient compositions (SnS_1−δ_, where δ = 0.05–0.075) using solid-state melting (≈10^–6^ Torr) and spark plasma sintering (SPS). The SnS_0.925_ (δ = 0.075) sample exhibited optimal performance, achieving a *ZT* of ≈0.03 at 770 K (Figure S1). Subsequently, the SnS_0.905_Cl_0.02_ sample was selected as a matrix for further research, with chlorine doping (0.02) acting as an additional free electron source by substituting sulfur atoms. The effect of Cl doping was further enhanced in SnS_0.455_Se_0.45_Cl_0.02_ due to the higher Se content, which lowers the band gap and facilitates Cl-induced carrier conduction (Figure 1b). Finally, we maintained a fixed Se content for SnS_0.925_ + *x* mol % SnCl_2_ samples to compensate for Sn vacancies and provide Cl for charge compensation. These modifications reduced lattice thermal conductivity (Figure 1c) and maintained electron transport properties. The cumulative effect resulted in a high thermoelectric figure of merit of approximately 0.7 at 823 K for n-type SnS_0.925_ + 0.03 mol % SnCl_2_.

**Figure 1:**
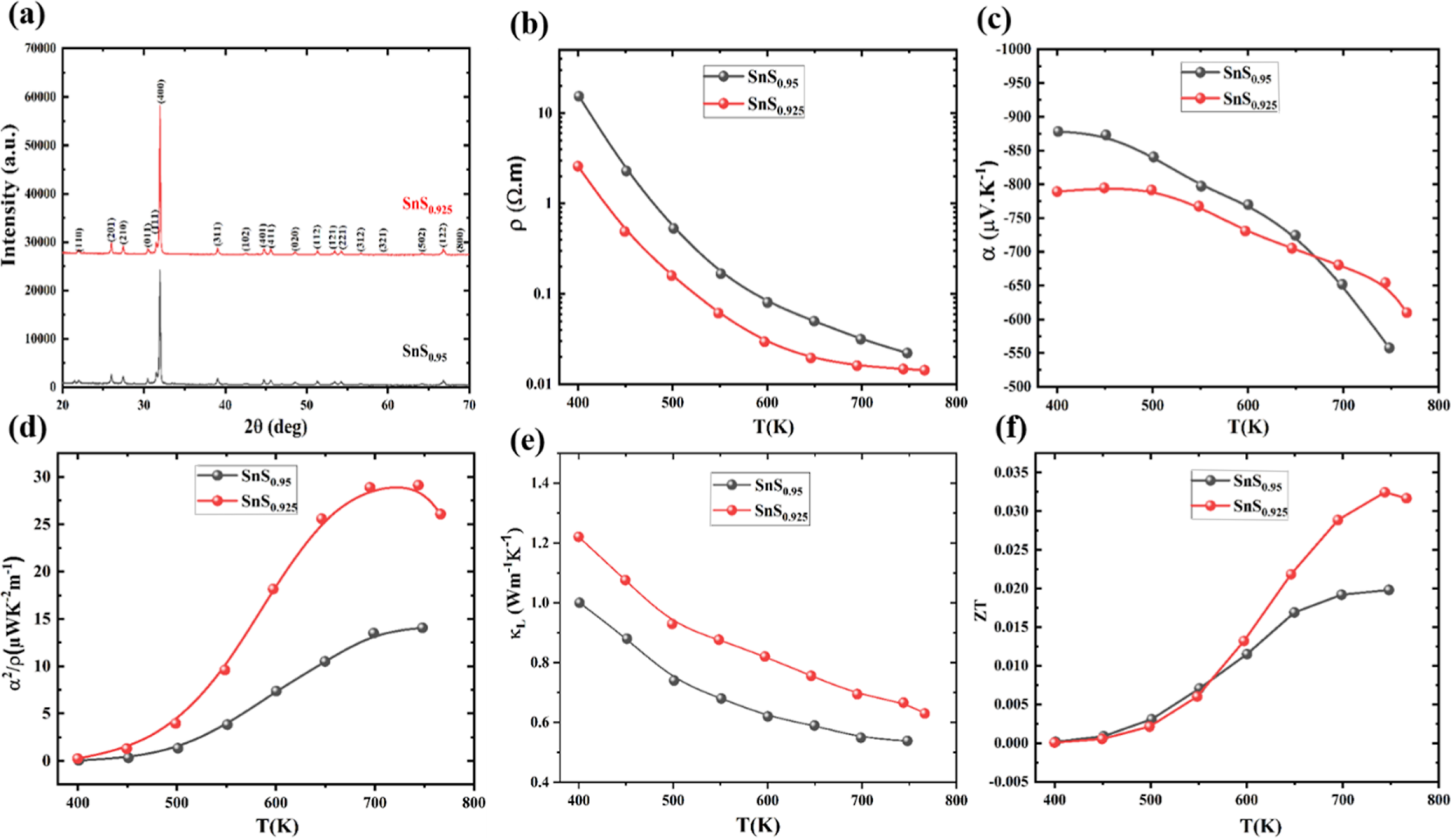
(a) XRD patterns of SnS_1−δ_ (*X* = 0.05, 0.075) samples, thermoelectric transport performance of n-type polycrystal SnS_1−δ_ samples (*X* = 0.05, 0.075). (b) Electrical resistivity. (c) Seebeck coefficient. (d) Power factor. (e) Lattice and electronic thermal conductivity. (f) Figure of merit (*ZT*) value.

## 2 Results and Discussion

### 2.1 Intrinsic n-Type SnS Enabled by Sulfur Vacancies

Figure 1 summarizes the structural and thermoelectric transport properties of sulfur-deficient SnS_1−δ_ (δ = 0.05 and 0.075). The XRD patterns (Figure 1a) confirm that both compositions crystallize in the orthorhombic SnS phase. To better visualize weak reflections and assess phase purity in the presence of preferred orientation, the corresponding log-scale XRD patterns and Rietveld refinement results are provided in Figures S1 and S2b. The temperature-dependent electrical resistivity (Figure 1b) decreases continuously with increasing temperature, consistent with semiconducting transport. Compared with SnS_0.95_, SnS_0.925_ exhibits substantially lower resistivity across the measured temperature range, reflecting a higher electron concentration (−6.77 × 10^15^ cm^–3^) introduced by sulfur vacancies acting as donor defects.^24,25,38^ The Seebeck coefficient (Figure 1c) are negative for both samples, confirming n-type conduction. The magnitude of S is moderately reduced for SnS_0.925_, consistent with its increased carrier density, as shown in Table S2. The combination of electrical resistivity and Seebeck coefficient led to a significant enhancement in the power factor for SnS_0.925_ (Figure 1d), which reaches a maximum near 700 K and exceeds that of SnS_0.95_ over the entire temperature range. As shown in Figure 1e, the lattice thermal conductivity decreases with increasing temperature for both samples due to intensifies phonon–phonon scattering. As a result, SnS_0.925_ achieves maximum *ZT* of 0.03 achieved at 770 K (Figure 1f), demonstrating that sulfur deficiency induces donor defects and provides an effective pathway to achieve and stabilize n-type conduction. Having established n-type SnS through S vacancy engineering, further enhancement of thermoelectric performance requires increasing the electron concentration, which is realized here by Cl and Se codoping in SnS_0.925–*x*_Cl_0.02_Se_*x*_.

### 2.2 Carrier Density Optimization in n-Type SnS_0.925_

#### 2.2.1 Structural Analysis of SnS_0.925–*x*_Cl_0.02_Se_*x*_

Figure 2a presents room-temperature X-ray diffraction (XRD) patterns of SnS_0.905–*x*_Se_*x*_Cl_0.02_ (*x* = 0.25, 0.35, 0.45) samples. The observed diffraction peaks align precisely with standard JCPDS card data, confirming the formation of an orthorhombic layered structure with *Pnma* space group symmetry. With increasing selenium content, the prominent (400) diffraction peak systematically shifts toward lower 2θ values (Figure 2b), indicating lattice expansion, as shown in Figure 2c. The sample without Se (SnS_0.905_Cl_0.02_) exhibits phase-pure characteristics with no detectable impurities. In contrast, selenium-containing samples (*x* = 0.25–0.45) display an additional diffraction peak at 2θ = 32.01°, denoted by an asterisk in Figure 2a. This peak, matching the diffraction pattern of elemental Sn, suggests secondary phase formation. The predominant intensity of the (400) peak reveals the preferential growth orientation of the samples. Rietveld refinement analysis of the SnS sample’s XRD pattern is illustrated in Figure S5.

**Figure 2:**
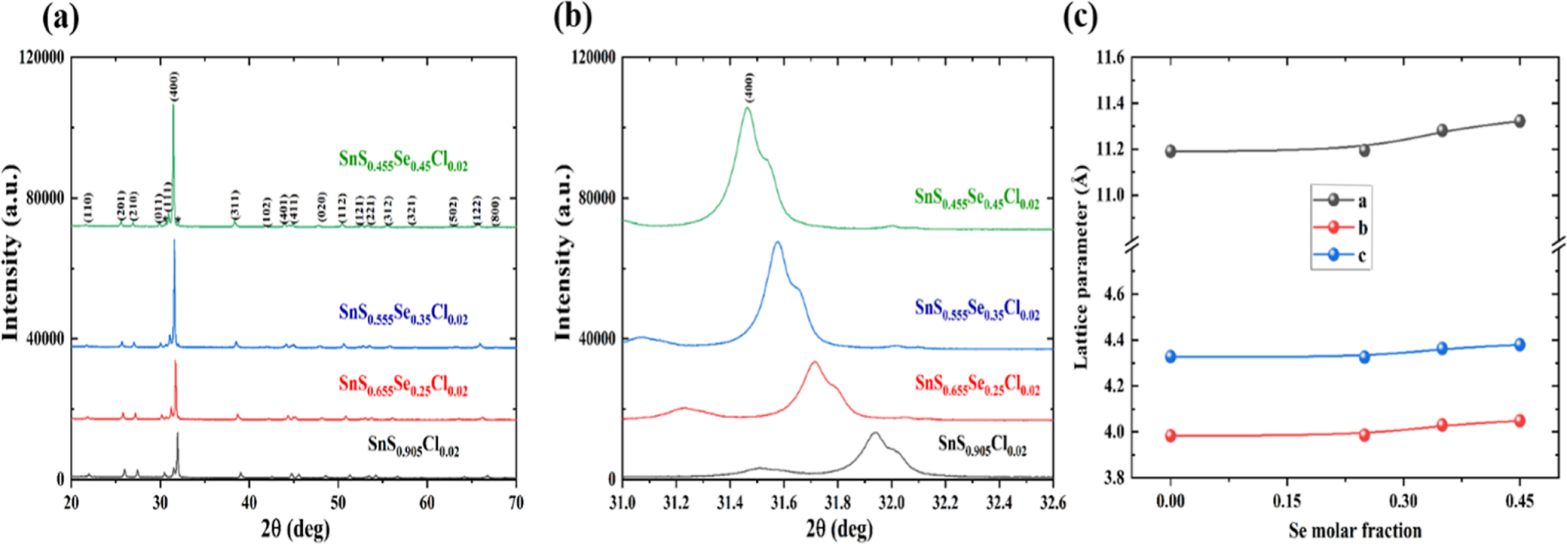
(a) XRD patterns of SnS_1–*x*_Se_*x*_Cl_0.02_ samples, asterisk symbols indicate the Sn impurities, which are prominent in *x* = 0.35 composition (b) closed view of (400) peak of SnS_1–*x*_Se_*x*_Cl_0.02_ samples, and (c) calculated lattice parameters for SnS_1–*x*_Se_*x*_Cl_0.02_ samples (*x* = 0.25, 0.35, 0.45).

#### 2.2.2 Electric Transport Properties

Figure 3a depicts the temperature-dependent electrical resistivity measurements of SnS_0.905–*x*_Se_*x*_Cl_0.02_ (*x* = 0.25, 0.35, 0.45) samples conducted along the parallel to the pressing direction. The temperature-dependent behavior demonstrates characteristic semiconducting transport properties across all compositions. The systematic incorporation of selenium results in a marked reduction in electrical resistivity compared to the selenium-free SnS_0.905_Cl_0.02_ sample. Among the studied compositions, SnS_0.455_Se_0.45_Cl_0.02_ exhibits lower resistivity. Specifically, at 308 K, SnS_0.455_Se_0.45_Cl_0.02_ achieves a minimal resistivity of 0.0049 Ω·m, representing an order of magnitude improvement over SnS_0.905_Cl_0.02_ (0.034 Ω·m). This enhanced electrical performance can be attributed to two synergistic effects: (i) the band gap reduction induced by selenium substitution and (ii) the improved activation of chlorine dopants. The combined effect manifests as a significant increase in carrier concentration from −2.66 × 10^18^ cm^–3^ in SnS_0.905_Cl_0.02_ to −1.48 × 10^19^ cm^–3^ in SnS_0.455_Se_0.45_Cl_0.02_ at room temperature, demonstrating successful carrier density optimization through compositional engineering. The activation energies derived from temperature-dependent resistivity measurements using the Arrhenius equation: $\rho \left(T\right)=\rho_{0}.e^{\left(E_{a}/K_{B}T\right)}$ , were found to be significantly lower than half of the transport band gaps (*E*_a_ ≪ *E*_g_/2), estimated via the Goldsmid–Sharp relation: *E*_g_ = 2*e*|*S*_max_|*T*_max_, as shown in Table S2. This observation confirms extrinsic conduction behavior. In SnS_0_._905_Cl_0_._02_, where specific values of *E*_a_ and *E*_g_ are observed, charge transport is attributed to thermally activated electrons originating from shallow donor levels, likely introduced by Cl substituting sulfur. For Se-substituted samples, the activation energy systematically decreases from 0.102 eV in SnS_0.655_Se_0.25_Cl_0.02_ to 0.069 eV in SnS_0.455_Se_0.45_Cl_0.02_. This reduction in activation energy, coupled with lower electrical resistivity, indicates an increase in carrier concentration. This trend suggests that Se incorporation modifies the defect structures, potentially increasing donor-like defects such as Se on S, thereby generating more free electrons and promoting n-type conduction at lower temperatures. Overall, transport in all samples is predominantly governed by thermally activated extrinsic electrons from shallow donor levels, as opposed to intrinsic excitation across the band gap.

**Figure 3:**
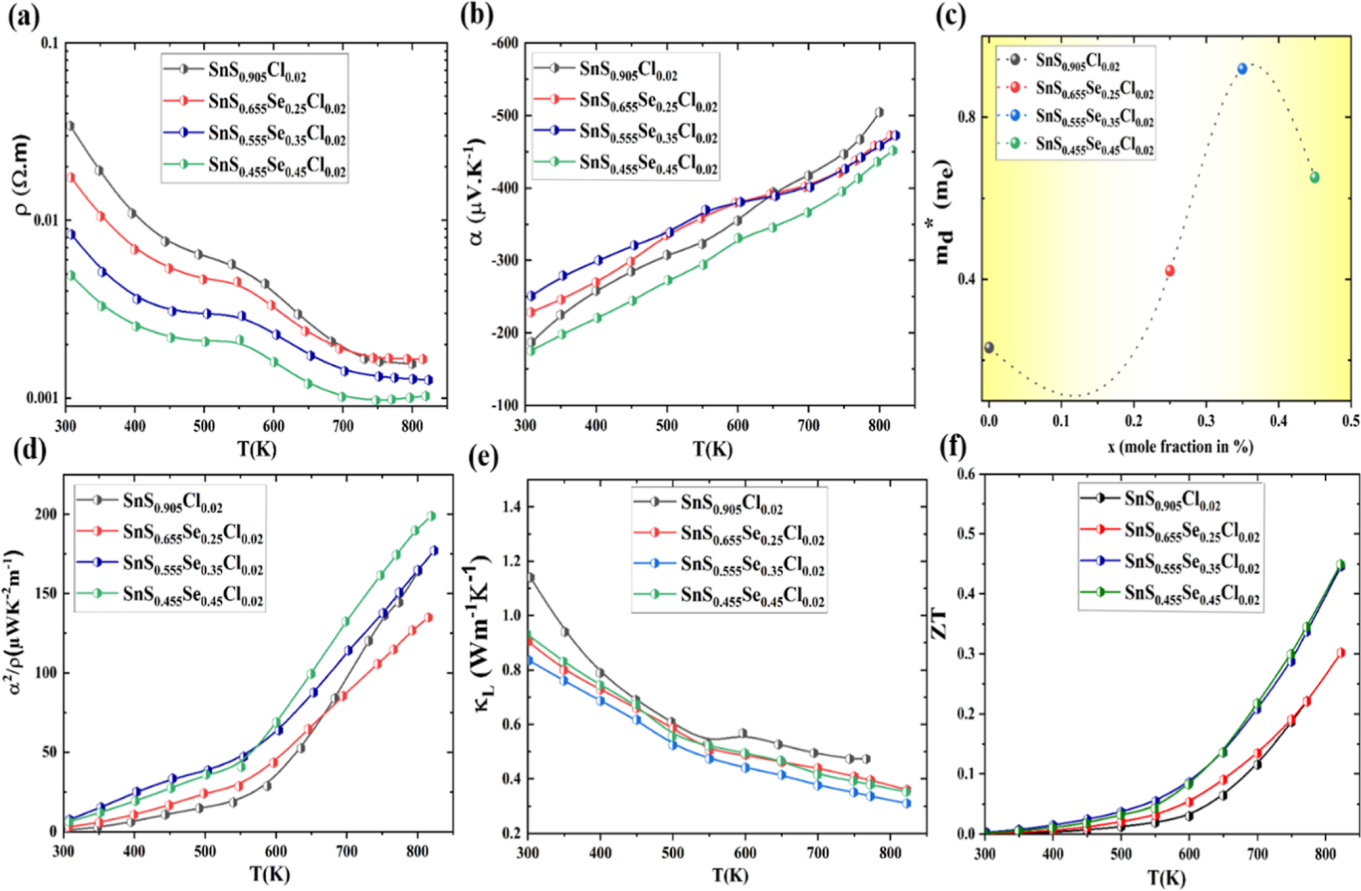
Thermoelectric transport performance of n-type polycrystal SnS_1–*x*_Se_*x*_Cl_0.02_ samples (*x* = 0.25, 0.35, 0.45). (a) Electrical resistivity. (b) Seebeck coefficient. (c) Density of states effective mass estimated from eq 1 at 308 K. (d) Power factor. (e) Lattice thermal conductivity. (f) Figure of merit (*ZT*) value.

The temperature-dependent Seebeck coefficient of SnS_0.905–*x*_Se_*x*_Cl_0.02_ (*x* = 0.25, 0.35, 0.45) samples, as shown in Figure 3b. The Seebeck coefficient (*S*) is inversely proportional to the carrier concentration. It follows Mott law $\left(S=\frac{\pi^{2}K_{B}^{2}}{3q}{\left[\frac{1}{n}\frac{\partial \left(E\right)}{\partial E}+\frac{1}{\mu }\frac{\partial \mu \left(E\right)}{\partial E}\right]}_{E=E_{f}}\right)$ , and it is also influenced by the effective mass of the carriers and the density of states near the Fermi level. Substitution with isovalent (Se^2–^) and aliovalent (Cl^–^) elements could alter band structures, leading to modified electronic transport properties. When engineering bands to enhance thermoelectric performance, the density of state effective mass (*m*_d_*) serves as a key indicator for band modification. *m*_d_* calculations, derived from Hall effect measurements and experimental Seebeck coefficient (*S*) data.^39,40^ It provides critical insights into how doping affects both *S* and electrical resistivity, ultimately influencing thermoelectric efficiency.

1 $$m_{d}^{*}=0.857\left(\frac{300\;K}{T}\right)m_{e}{\left(\frac{n}{10^{20}{\;cm}^{-3}}\right)}^{2/3}\left[\frac{3{\left(\exp\left[\frac{\left|S\right|}{K_{B}/e}-2\right]-0.17\right)}^{2/3}}{1+\exp\left[-5\left(\frac{\left|S\right|}{K_{B}/e}-\frac{K_{B}/e}{\left|S\right|}\right)\right]}+\frac{\frac{\left|S\right|}{K_{B}/e}}{1+\exp\left[(\frac{\left|S\right|}{K_{B}/e}-\frac{K_{B}/e}{\left|S\right|})\right]}\right]$$

where *m*_d_* is the density of state effective mass, *m*_e_ is the rest mass of an electron, n is the carrier concentration, *K*_B_ is the Boltzmann constant, *e* is the charge of the electron, and *S* is the Seebeck coefficient. The density of state effective mass (*m*_d_*) varies with Se­(*x*) content in SnS_0.905_Cl_0.02_, as illustrated in Figure 3c. At room temperature, SnS_0.655_Se_0.25_Cl_0.02_ and SnS_0.555_Se_0.35_Cl_0.02_ compositions demonstrate enhanced S values relative to SnS_0.905_Cl_0.02_. This enhancement correlates with the increased *m*_d_* values, which rise from 0.231 *m*_e_ in SnS_0.905_Cl_0.02_ to 0.421 *m*_e_ in SnS_0.655_Se_0.25_Cl_0.02_ and 0.919 *m*_e_ in SnS_0.555_Se_0.35_Cl_0.02_. In contrast, the SnS_0.455_Se_0.45_Cl_0.02_ composition exhibits a lower *S* than compositions with lower Se content. This reduction is attributed to its increased carrier density and lower density of state effective mass (*m*_d_* = 0.651 *m*_e_). However, these characteristics contribute to increased carrier density, resulting in an enhanced power factor of ≈198.89 μWm^–1^ K^–2^ at 820 K SnS_0.455_Se_0.45_Cl_0.02_, as shown in Figure 3d.

Figure 3e illustrates the temperature-dependent thermal conductivity of SnS_0.905–*x*_Cl_*x*_ (*x* = 0.25–0.45) samples measured parallel to the pressing direction. The lattice thermal conductivity (κ_l_) consistently decreases with increasing temperature across all Se­(*x*)-substituted compositions. Notably, the SnS_0.555_Se_0.35_Cl_0.02_ composition exhibits an optimal lattice thermal conductivity of 0.30 Wm^–1^ K^–1^ at 822 K, surpassing the performance of the higher Se-substituted SnS_0.455_Se_0.45_Cl_0.02_ composition (κ_l_ ≈ 0.35 Wm^–1^ K^–1^ at 822 K). This superior thermal behavior in SnS_0.555_Se_0.35_Cl_0.02_ can be attributed to its optimized ratio of Sn–S (S ∼ 100 pm) to Sn–Se (Se ∼ 120 pm) bonds, which induces lattice anharmonicity distortion and strain, thereby enhancing phonon scattering efficiency. Moreover, the presence of SnCl_2_ and Sn precipitates further enhances phonon scattering, leading to a reduction in lattice thermal conductivity. The electronic contribution to thermal conductivity (κ_ele_) was evaluated using the Wiedemann–Franz relationship κ_ele_ = *L*σ*T*, where *L* represents the Lorenz constant (detailed in Figure S6, Supporting Information). Analysis reveals exceptionally low κ_ele_ values, demonstrating its negligible contribution to the total thermal conductivity (κ_tot_). This finding indicates that κ_tot_ is primarily dominated by lattice thermal conductivity (κ_lat_), a characteristic that suggests the carrier concentration (*n*) remains suboptimal. The current carrier concentration state presents substantial opportunities for enhancement through further materials engineering, doping, and vacancy compensation strategies. As illustrated in Figure 3f, a significant enhancement in thermoelectric figure of merit (*ZT*) was achieved, increasing from ≈0.22 in SnS_0.905_Cl_0.02_ at 773 K to ≈0.45 in SnS_0.455_Se_0.45_Cl_0.02_ at 823 K. This substantial improvement can be attributed to the synergistic effect of enhanced power factor (PF) and moderate total thermal conductivity (κ_tot_).

### 2.3 SnS_0.475_Se_0.45_ Vacancy Compensation

The thermoelectric performance of n-type polycrystalline SnS_0.455_Se_0.45_Cl_0.02_ was optimized by strategically incorporating SnCl_2_, which effectively compensates for intrinsic Sn vacancies. Under S-poor conditions, despite S exhibiting lower formation energy compared to Sn vacancies, it proves ineffective in compensating for hole carriers generated by Sn vacancies.^24,25^ This behavior is attributed to the energetic positioning of both Sn vacancy states and Sn_*i*_ transition levels, which lie in closer proximity to the valence band maximum relative to the conduction band minimum.^24–26^ Consequently, residual Sn vacancies play a crucial role in determining the electronic properties of n-type SnS polycrystals, even under S-poor synthesis conditions. The formation energy of Sn vacancies increased under Sn-rich conditions compared to Sn-poor conditions. This higher formation energy indicates the reduced probability of Sn vacancy formation following Sn addition. These results demonstrate that incorporating excess Sn compensates for intrinsic Sn vacancies, thereby reducing the overall Sn vacancy concentration.^24–26^ The XRD patterns of SnS_0.475_Se_0.45_ samples with varying SnCl_2_ content (*x* = 0.01, 0.02, 0.03 mol %) are presented in Figure 4a. All samples exhibit primary diffraction peaks consistent with the SnS phase, alongside additional Sn-related diffraction peaks. With increasing SnCl_2_ concentration, the diffraction peaks initially shift to lower angles (at 0.01 and 0.02 mol % SnCl_2_) and subsequently to higher angles (at 0.03 mol % SnCl_2_), as shown in Figure 4b. This systematic peak shift can be attributed to the initial substitution of Sn^2+^ ions at lattice sites. At higher concentrations, excess Sn preferentially forms Sn–Se bonds, driven by their significant electronegativity difference. The addition of SnCl_2_ partially results in the formation of SnCl_2_ along with Sn impurity phases, which impacts thermal transport. Rietveld refinement analysis of the SnS sample’s XRD pattern is illustrated in Figure S7. The role of SnCl_2_ addition should be understood in terms of defect-equilibrium modification rather than idealized full lattice substitution.^24,25^ SnCl_2_ does not fully dissolve into the SnS lattice as a simple substitutional defect complex. Instead, its addition increases the Sn chemical potential while introducing Cl^–^ donors. The elevated Sn chemical potential raises the formation energy of Sn vacancies (V_Sn_), thereby suppressing their formation, while Cl^–^ substitution on S sites provides electronic doping.^24,25^ Excess Sn or SnCl_2_ beyond the solubility limit forms isolated secondary phases, as confirmed by SEM–EDS mapping (Figures S8 and S9), rather than contributing to substitutional defects. This defect-equilibrium shift underlies the simultaneous suppression of Sn vacancies and control of electrical conductivity.

**Figure 4:**
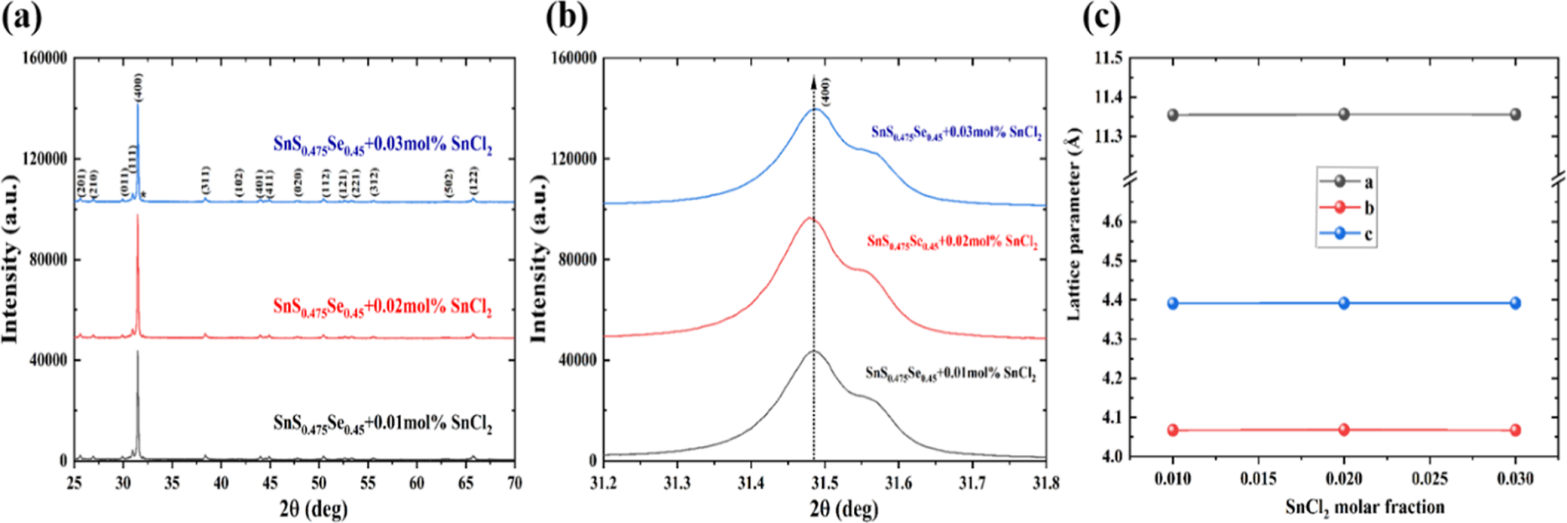
(a) XRD patterns of SnS_0.475_Se_0.45_ + *x* mol % SnCl_2_ samples, (b) closed view of (400) peak of SnS_0.475_Se_0.45_ + *x* mol % SnCl_2_ samples, and (c) calculated lattice parameters for SnS_0.475_Se_0.45_ + *x* mol % SnCl_2_ samples (*x* = 0.01, 0.02, 0.03).

Figure 5a illustrates the temperature-dependent electrical resistivity measurements of SnS_0.475_Se_0.45_ + *x* mol % SnCl_2_ (*x* = 0.01, 0.02, 0.03) samples along the parallel direction. All samples exhibit decreasing electrical resistivity with increasing temperature, confirming semiconductor behavior. The resistivity anomaly observed at 550 K in n-type SnS, while the actual phase transition occurs at 880 K, suggests the presence of other influencing factors. This anomaly could be attributed to enhanced defect states, such as increased activation of Sn or S vacancies, which temporarily trap charge carriers and increase resistivity. Additionally, increased carrier scattering due to impurity phases or subtle structural rearrangements might contribute to this behavior. Despite the rise in resistivity, the Seebeck coefficient remains unaffected, indicating stable carrier concentration and energy distribution. The incorporation of SnCl_2_ systematically increases the electrical resistivity at 300 K from 0.003 Ω·m (*x* = 0.01) to 0.004 Ω·m (*x* = 0.02) and 0.005 Ω·m (*x* = 0.03). This trend correlates with a concurrent decrease in carrier concentration and carrier mobility from −6.61 × 10^18^ cm^–3^ & 2.742 cm^2^ v^–1^ s^–1^ (*x* = 0.01) to −5.41 × 10^18^ cm^–3^ & 2.643 cm^2^ v^–1^ s^–1^ (*x* = 0.02) and −3.79 × 10^18^ cm^–3^ & 2.334 cm^2^ v^–1^ s^–1^ (*x* = 0.03) at 308 K, as shown in Figure 4b. Moreover, across all SnS_0.475_Se_0.45_ + *x* mol % SnCl_2_ (*x* = 0.01, 0.02, 0.03) samples were shown the activation energy was significantly lower than half the Goldsmid–Sharp band gap, confirming conduction via extrinsic donor levels. A shallow activation energy of 0.055 eV at 0.01 mol % SnCl_2_ indicates efficient thermal ionization of dopants. However, increasing the doping level to 0.03 mol % SnCl_2_ resulted in a rise in activation energy to 0.079 eV, suggesting either deeper donor states or potential compensation effects. These observations indicate an optimal dopant range of 0.01–0.02 mol % SnCl_2_ for maximizing carrier activation without degrading electronic transport. Furthermore, the 0.03 mol % SnCl_2_ sample exhibits higher electrical resistivity, likely due to dopant oversaturation. This oversaturation can lead to defect compensation, such as the formation of charge-neutral Cl–V_Sn_ complexes, and clustering of SnCl_2_ atoms. Such phenomena reduce the effective donor concentration and increase carrier scattering, thereby limiting free electron generation and degrading conductivity despite the increased nominal doping.

**Figure 5:**
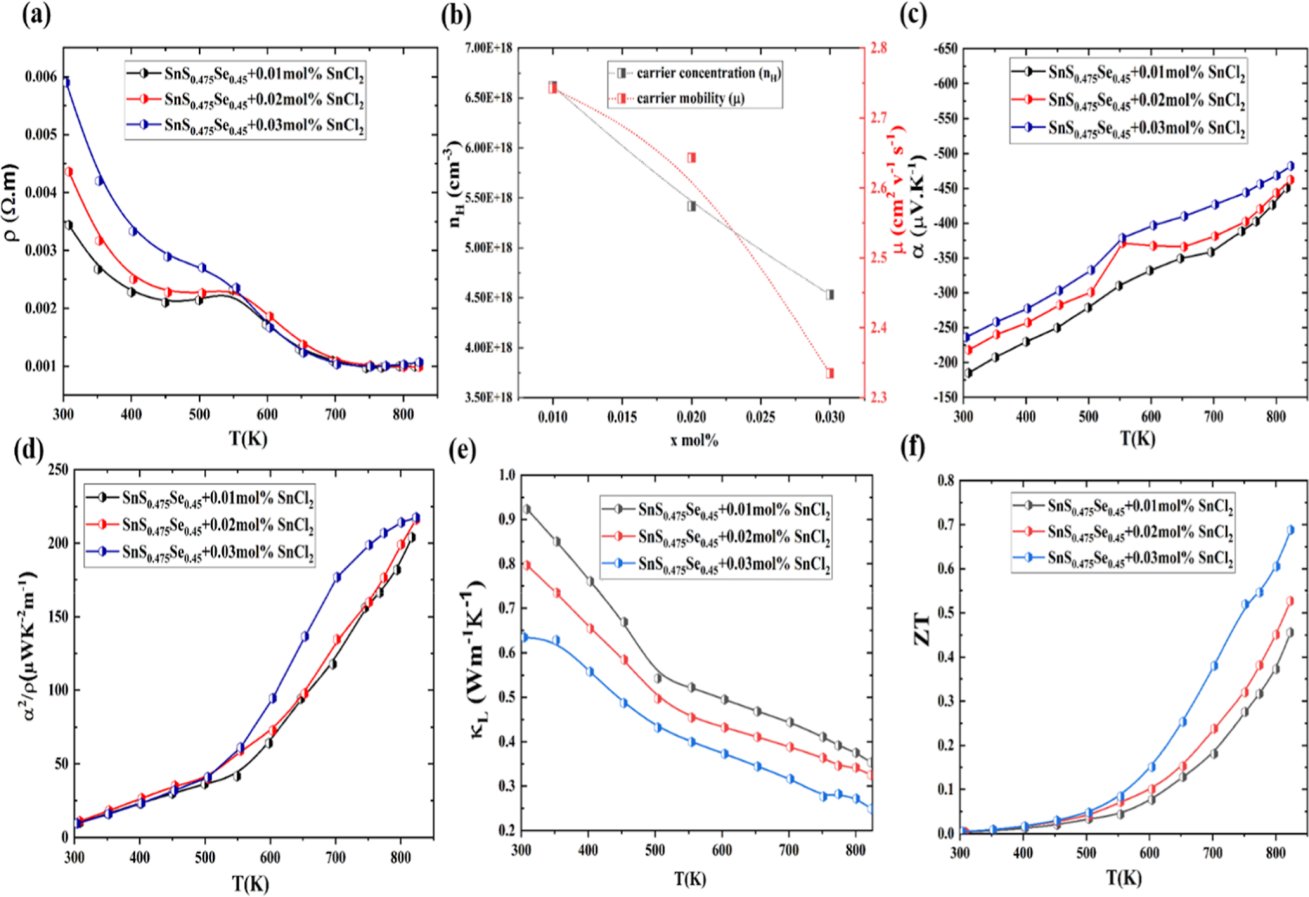
Thermoelectric transport performance of n-type polycrystal SnS_0.475_Se_0.45_ + *x* mol % SnCl_2_ samples (*x* = 0.01, 0.02, 0.03). (a) Electrical resistivity. (b) Carrier concentration and mobility at 308 K. (c) Seebeck coefficient. (d) Power factor. (e) Lattice thermal conductivity. (f) *ZT* value.

Furthermore, the Seebeck coefficient (*S*) of n-type polycrystalline SnS_0.475_Se_0.45_ + *x* mol % SnCl_2_ samples shows systematic enhancement with increasing SnCl_2_ content, consistent with the observed carrier concentration trends (Figure 5b,c). The optimized interplay between electrical resistivity and the Seebeck coefficient results in an enhanced power factor of ≈217 μWm^–1^ K^–2^ at 823 K (Figure 5d). The temperature-dependent lattice thermal conductivity of SnS_0.475_Se_0.45_ + *x* mol % SnCl_2_ samples was measured from 308 to 823 K, as shown in Figure 5e. The SnS_0.475_Se_0.45_ + 0.03SnCl_2_ sample exhibited a low lattice thermal conductivity of 0.63 Wm^–1^ K^–1^ at 300 K, which decreased to 0.24 Wm^–1^ K^–1^ at 823 K, lower than previously reported values.^27,28,41^ The reduced *K*_l_ in the SnS_0.475_Se_0.45_ + 0.03SnCl_2_ sample is attributed to solid solution point defects and secondary phase precipitates, creating more scattering centers that effectively impede phonon transport. This leads to a significant reduction in *K*_tot_ (approximately 0.26 Wm^–1^ K^–1^ at 823 K), as shown in Figure S10. The combined effect of improved power factor (PF) and low lattice thermal conductivity results in an impressive *ZT* value of ≈0.7 at 823 K for the SnS_0.475_Se_0.45_ + 0.03SnCl_2_ sample, as shown in Figure 5f.

To quantify the intrinsic electrical transport properties of SnCl_2_-doped SnS_0.925_, we calculated the weighted mobility (μ_w_) using

2 $$\mu_{w}=\frac{3h^{3}\sigma }{8\pi e{\left(2m_{e}K_{B}T\right)}^{3/2}}\left[\frac{\exp\left[\frac{\left|S\right|}{K_{B}/e}-2\right]}{1+\exp\left[-5\left(\frac{\left|S\right|}{K_{B}/e}-1\right)\right]}+\frac{\frac{3}{\pi^{2}}\frac{\left|S\right|}{K_{B}/e}}{1+\exp\left[5\frac{\left|S\right|}{K_{B}/e}-1\right]}\right]$$

Among all compositions, the SnS_0.925_ + 0.03 mol % SnCl_2_ sample demonstrated the highest μ_w_, as shown in Figure 6a, which confirms its enhanced electrical transport properties. The μ_w_/κ_l_ ratios of SnS_0.925_ + *x* mol % SnCl_2_ consistently surpassed those of undoped SnS_0.925_ throughout the measured temperature range (Figure 6b), demonstrating that SnCl_2_ doping successfully optimizes both electron and phonon transport mechanisms. To demonstrate the decoupling of electrical and thermal transport properties in n-type SnS polycrystals, we calculated the quality factor, $B={\left(\frac{k_{B}}{e}\right)}^{2}\frac{8\pi e{\left(2m_{e}k_{b}T\right)}^{3/2}}{3h^{3}}\cdot \frac{\mu_{w}}{k_{L}}\cdot \left(T\right)$ . Since quality factor *B* is linearly proportional to μ_w_/κ_L_, both parameters showed significant improvement in the SnS_0.475_Se_0.45_ + 0.03 mol % SnCl_2_ sample, as shown in Figure 6c. Moreover, *K*_l_ of SnS_0.475_Se_0.45_ + 0.03 mol % SnCl_2_ sample was largely reduced to an ultralow value of ∼0.24 Wm^–1^ K^–1^ at 823 K, which is relatively lower than other reported values,^27,28,41^ as shown in Figure 6c. The figure of merit is a crucial parameter for evaluating the performance of thermoelectric materials, and its value is temperature-dependent. Since these materials typically operate across various temperatures, calculating the average *ZT* becomes essential. The *ZT*_ave_ is calculated using the following formula

3 $${ZT}_{ave}=\frac{1}{T_{h}-T_{c}}\int_{T_{c}}^{T_{h}}ZT\;dT$$

**Figure 6:**
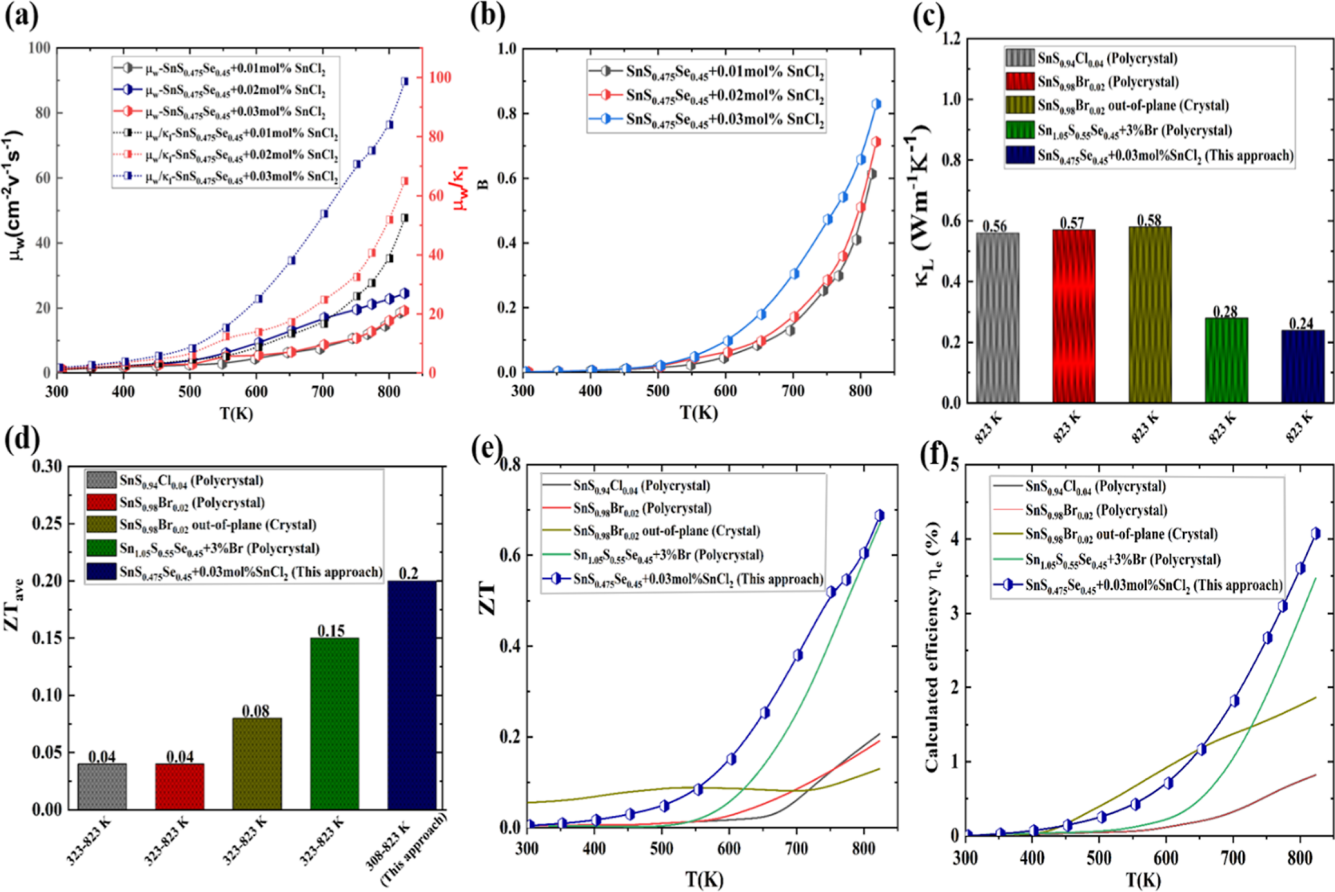
(a) Temperature-dependent weighted mobility (μ_w_) and (μ_w_/*K*_l_) of polycrystalline SnS_0.475_Se_0.45_ + *x* mol % SnCl_2_ samples (*x* = 0.01, 0.02, 0.03). (b) Quality factor. (c) Comparison of lattice thermal conductivity for n-type polycrystal SnS_0.475_Se_0.45_ + 0.03SnCl_2_ samples in this study and other n-type SnS.^27,28,41^ (d) Average *ZT* (*ZT*_avg_) comparison with reported polycrystalline n-type SnS.^27,28,41^ (e) comparison of *ZT* for n-type SnS_0.475_Se_0.45_ + 0.03SnCl_2_ samples with reported values.^27,28,41^ (f) Calculated efficiency (η_e_) with reported values.^27,28,41^

The calculated *ZT*_ave_ value of 0.2 for the SnS_0.475_Se_0.45_ + 0.03 mol % SnCl_2_ sample was obtained over the 308–823 K temperature range, as shown in Figure 6d. The theoretical thermoelectric conversion efficiency was then determined using the Synder approach

4 $$\eta_{e}=\frac{T_{h}-T_{c}}{T_{H}}\frac{\sqrt{1+{ZT}_{ave}}-1}{\sqrt{1+{ZT}_{ave}+\frac{T_{c}}{T_{h}}}}$$

where *T*_h_ and *T*_c_ are the temperatures of the hot side and cold side temperature of the TE module, respectively, with *T*_c_ fixed at 323 K. As shown in Figure 6e, the calculated maximum *ZT* efficiency reaches approximately 4.01% for SnS_0.475_Se_0.45_ + 0.03SnCl_2_ samples. These samples demonstrate superior thermoelectric performance across the entire temperature range compared to previously reported n-type SnS.^27,28,41^ Figure 6f shows enhanced *ZT* values in the 323–623 K range compared to polycrystalline SnS materials with single metal halide or elemental metal dopants.^27,28,41^ This innovative codoping strategy using metal halides and metal elements to optimize thermoelectric properties can be applied to other thermoelectric systems. The approach shows promising potential for thermoelectric applications in medium-temperature ranges.

## 3 Conclusion

In this study, we systematically enhanced the thermoelectric efficiency of n-type SnS through a multistep approach, achieving significant improvements in its figure of merit (*ZT*). By generating sulfur vacancies and implementing strategic dopants, including aliovalent Cl and isoelectronic Se, we effectively reduced the band gap and increased carrier concentration. This led to a notable *ZT* value of 0.45 at 823 K in SnS_0.455_Se_0.45_Cl_0.02_. Further optimization, addressing intrinsic Sn and S deficiencies through SnCl_2_ incorporation, yielded a remarkable peak *ZT* of 0.7 at 823 K in SnS_0.475_Se_0.45_ + 0.03 mol % SnCl_2_. The synergistic effects of enhanced phonon scattering and suppressed hole carrier generation resulted in a low thermal conductivity (κ_L_) of 0.24 W m^–1^ K^–1^. This comprehensive methodology not only underscores the potential of n-type SnS for high-performance thermoelectric applications but also establishes a robust framework for future advancements in SnS-based materials. The integration of metal chlorides within the SnS matrix opens new avenues for optimizing thermoelectric properties, paving the way for environmentally friendly and efficient energy conversion technologies.

## 4 Experimental Procedure

Polycrystalline SnS_1−δ_ (*X* = 0.05, 0.075), SnS_0.905–*x*_SeCl_0.02_ (*x* = 0.25, 0.35, 0.45), and SnSe_0.475_Se_0.45_ + *x* mol % SnCl_2_ (where *x* = 0.01, 0.02, 0.03) samples were meticulously synthesized through the combination of stoichiometric ratios of elemental Sn (99.999%), S (99.999%, Alfa Aesar), Se (99.999%) and powdered SnCl_2_ (99.99%) powder were vacuum sealed in a quartz tube. These tubes were then vacuum sealed at 10^–6^ Torr. The sealed tubes underwent heating to 1223 K over 15 h, maintained at 12 h, and then cooled down naturally. The synthesized ingots underwent a process of comminution using a mortar and pestle, followed by granulometric separation via sieving to yield a fine powder. This prepared powder was subsequently consolidated into dense compacts through spark plasma sintering (SPS), utilizing an SPS211-LX apparatus manufactured by Dr. Sinter Lab, in preparation for the characterization of thermoelectric properties. The finely divided samples were molded into cylindrical forms, exhibiting nominal dimensions of 12.7 mm in diameter and 8 mm in axial thickness, achieved through SPS processing employing graphite dies under an applied uniaxial pressure of 50 MPa at a sintering temperature of 570 °C. Postconsolidation density measurements (ρ) indicated a relative density of approximately 95% with respect to the theoretical maximum density. Concluding the preparation, the consolidated samples were meticulously sectioned and surface-treated through polishing along a predetermined parallel orientation, designed to enable precise measurement of anisotropic electrical and thermal transport properties inherent to the n-type SnS material.

### 4.1 Thermoelectric Property Measurement

Thermoelectric measurements were conducted in a parallel orientation relative to the direction of applied pressure during sample preparation. Electrical resistivity and Seebeck coefficient values were ascertained utilizing a ULVAC-Riko ZEM 3 apparatus, operating under conditions of low pressure and within a helium-enriched atmosphere, spanning a temperature interval from 300 to 823 K. Determination of carrier concentration was achieved through Hall effect measurements, performed on rectangular specimens consistent with those employed for electrical transport assessments within a Physical Property Measurement System (PPMS) at magnetic field strengths up to 0.9 T at ambient temperature. Thermal diffusivity (*D*) as a function of temperature was evaluated via a laser flash method, employing a Netzsch LFA-467 instrument. Subsequently, total thermal conductivity (κ) was derived from the relation κ = *D* × *C*_*p*_ × ρ, where *C*_*p*_ denotes specific heat capacity. Dimensional specifications for samples utilized in κ assessments were established at 12.7 mm in diameter and 2 mm in thickness, with a parallel alignment. The cumulative uncertainty associated with all measurement parameters contributing to determining the figure of merit (*ZT*) was maintained below a threshold of 15%.

## Supplementary Material



## Notes

Log scale XRD pattern, Rietveld refinement results, SEM–EDS characterization, lattice parameter table, and additional thermoelectric transport data (PDF)
