# Supplementary material for: Synergistically Optimizing the Thermoelectric Performance of n‑Type SnS through an Integrated Systematic Approach
Source: ACS Appl Mater Interfaces. 2026 Feb 18;18(8):12633–42. doi: 10.1021/acsami.5c21755 (PMC12964349; doi:10.1021/acsami.5c21755)
Supplement: Supplementary file 1 [file am5c21755_si_001.pdf]

Supporting Information

Synergistically optimizing the thermoelectric performance of n-type SnS through an integrated systematic approach

Sidharth Duraisamy,[a] Yang-Yuan Chen,[a] Kuei-Hsien Chen,[b] Maw-Kuen Wu,[a] G. Peramaiyan,[a] V. K. Ranganayakulu,[a] Muluken Biadgelegn Wollele,[a] Min-Nan Ou*[a]

[a] *Institute of Physics, Academia Sinica, Taiwan 115.*

[b] *Institute of Atomic and Molecular Sciences, Academia Sinica, Taiwan 115.*

**Corresponding author:* oumn@gate.sinica.edu.tw

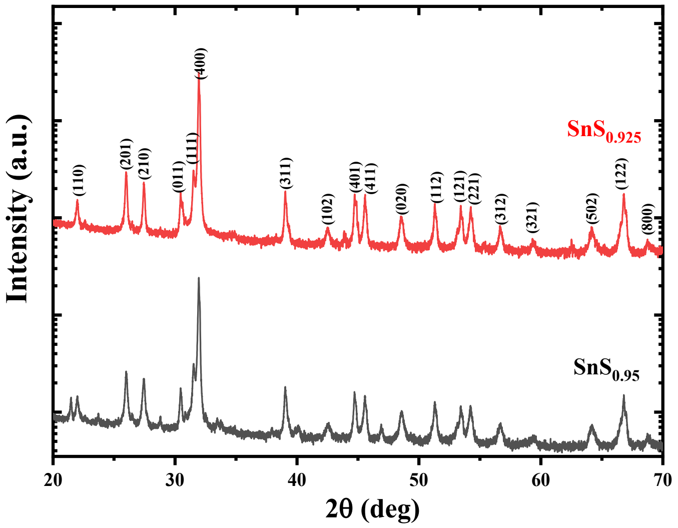

Figure S1. Log-scale XRD pattern of $SnS_{0.925}$, shown to reveal weak diffraction features that are not readily visible in linear-scale plots due to strong preferred orientation in layered SnS.

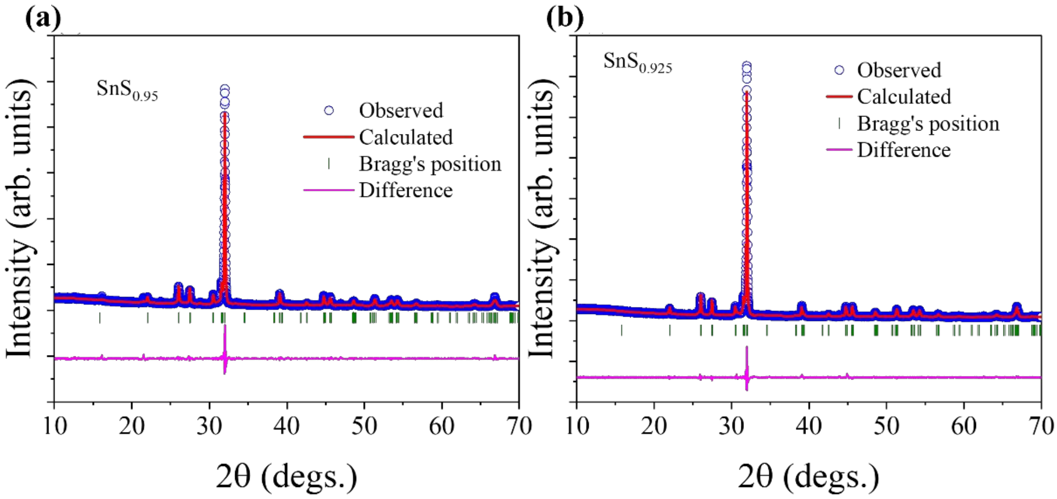

Fig. S2. XRD pattern with rietveld refinement of the $SnS_{1-\delta}$ (X=0.05, 0.075) samples

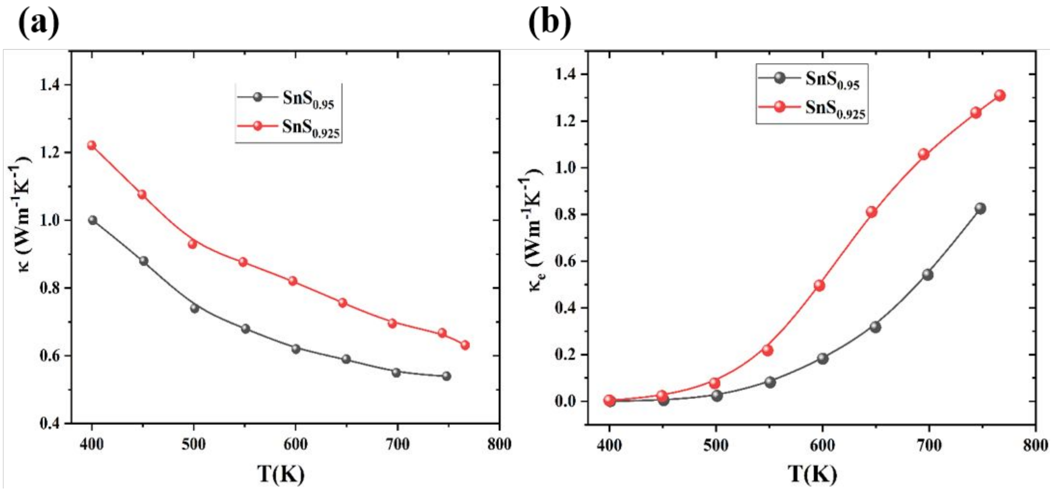

Fig. S3. (a&b) Total and electronic thermal conductivity of the $SnS_{0.905-x}Se_xCl_{0.02}$ (x=0.25, 0.35, 0.45).

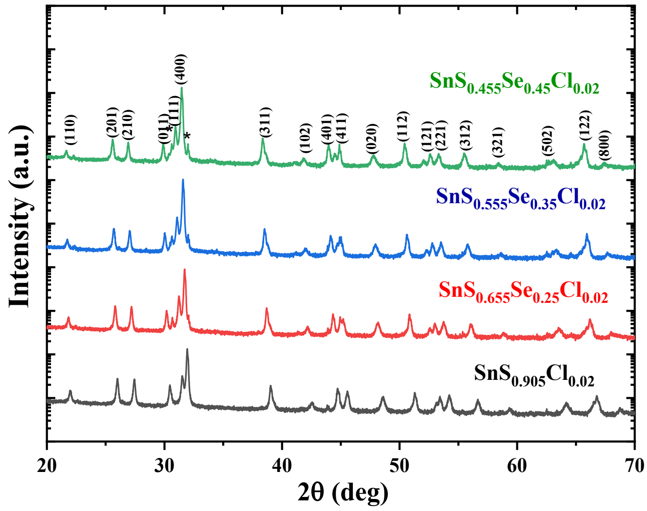

Figure S4. Log-scale XRD pattern of $SnS_{0.925}$, shown to reveal weak diffraction features that are not readily visible in linear-scale plots due to strong preferred orientation in layered SnS.

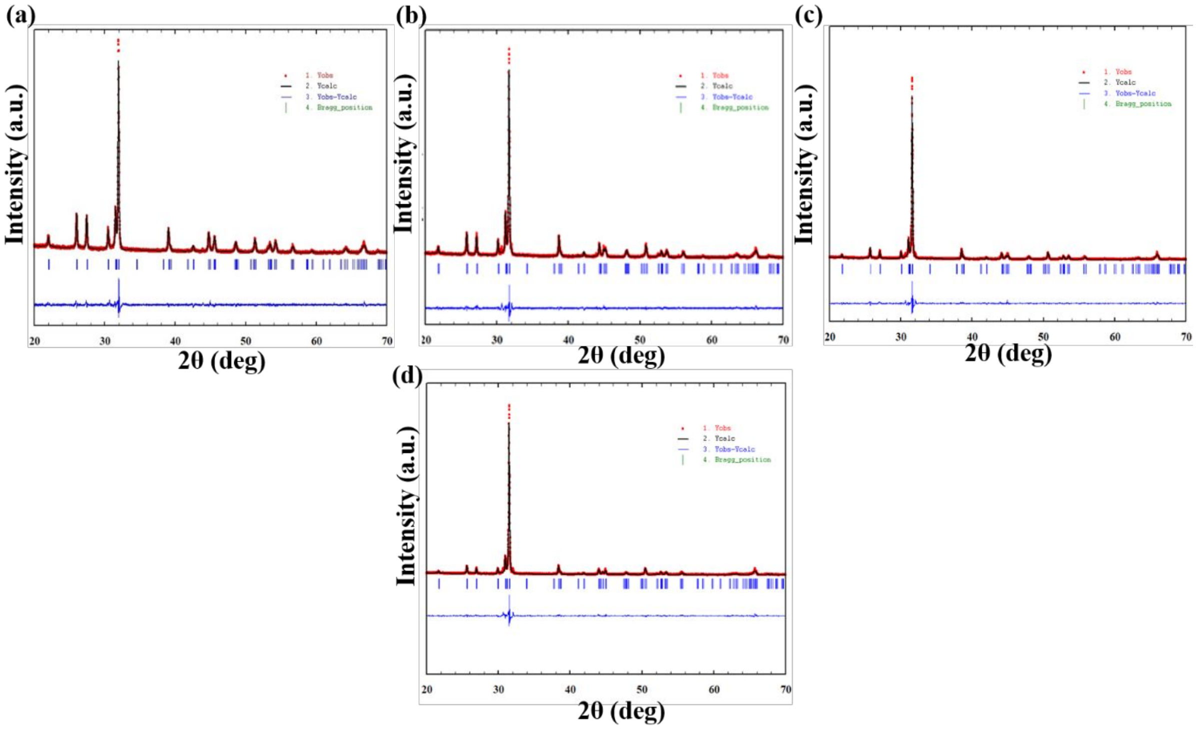

Fig. S5. XRD pattern with rietveld refinement of the $SnS_{0.905-x}Se_xCl_{0.02}$ **(x=0.25, 0.35, 0.45). (a)** $SnS_{0.905}Cl_{0.02}$**. (b)** $SnS_{0.655}Se_{0.25}Cl_{0.02}$**. (c)** $SnS_{0.555}Se_{0.25}Cl_{0.02}$**. (d)** $SnS_{0.455}Se_{0.25}Cl_{0.02}$**.**

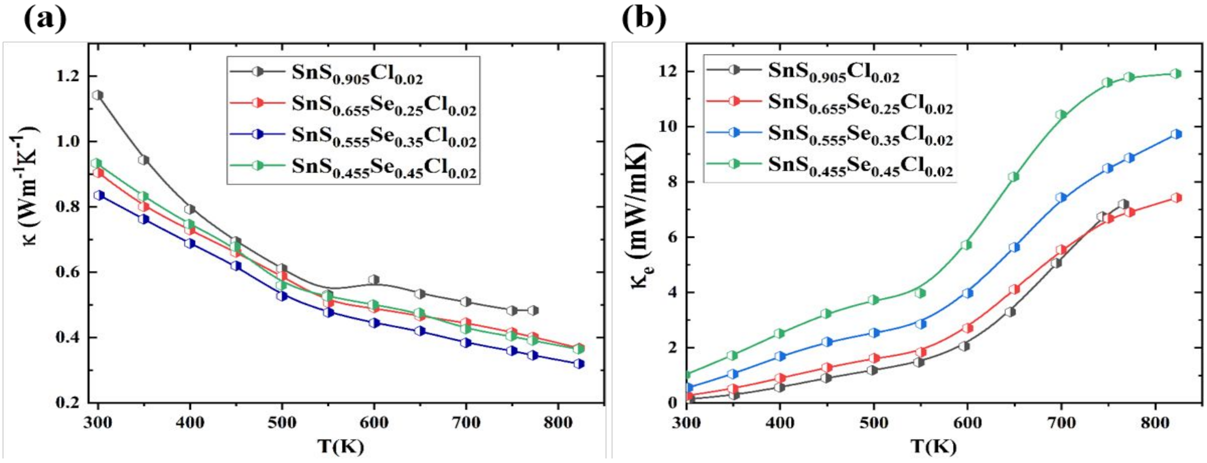

Fig. S6. (a&b) Total and electronic thermal conductivity of the $SnS_{0.905-x}Se_xCl_{0.02}$ **(x=0.25, 0.35, 0.45).**

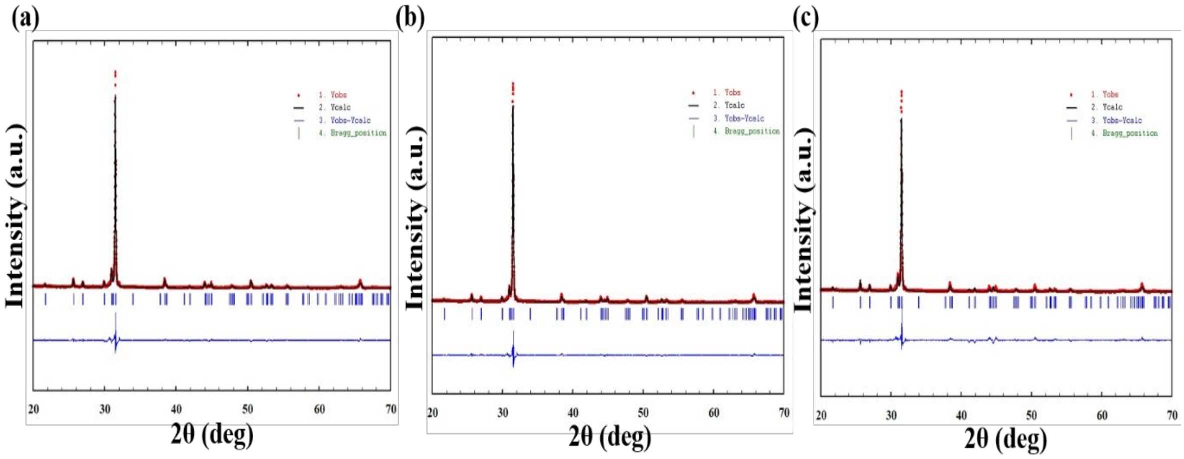

Fig. S7. XRD pattern with rietveld refinement of the $SnS_{0.475}Se_{45}$+xmol%$SnCl_2$ (x=0.01, 0.02, 0.03). (a) $SnS_{0.475}Se_{45}$+0.01$SnCl_2$. (b) $SnS_{0.475}Se_{45}$+0.02$SnCl_2$. (c) $SnS_{0.475}Se_{45}$+0.03$SnCl_2$.

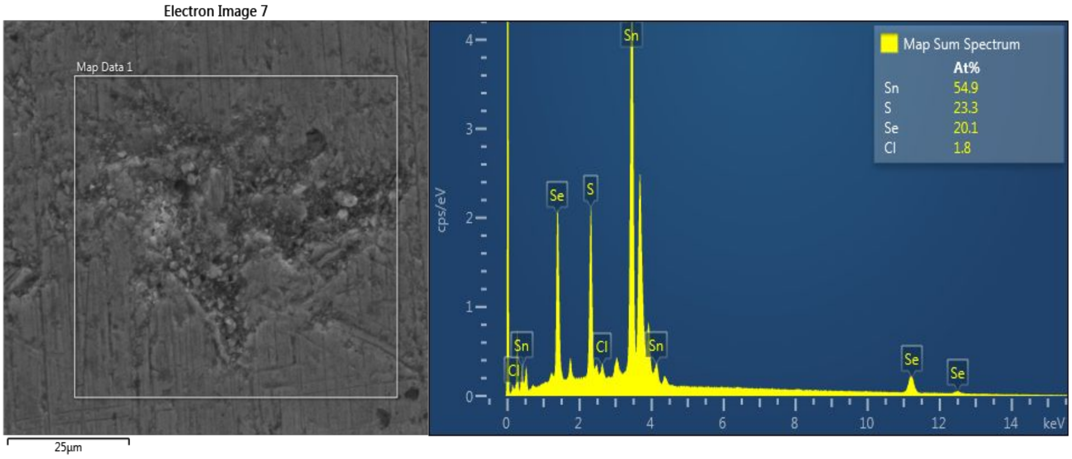

Fig. S8. SEM–EDS characterization of n-type $SnS_{0.475}Se_{0.45}$ + 0.03$SnCl_2$

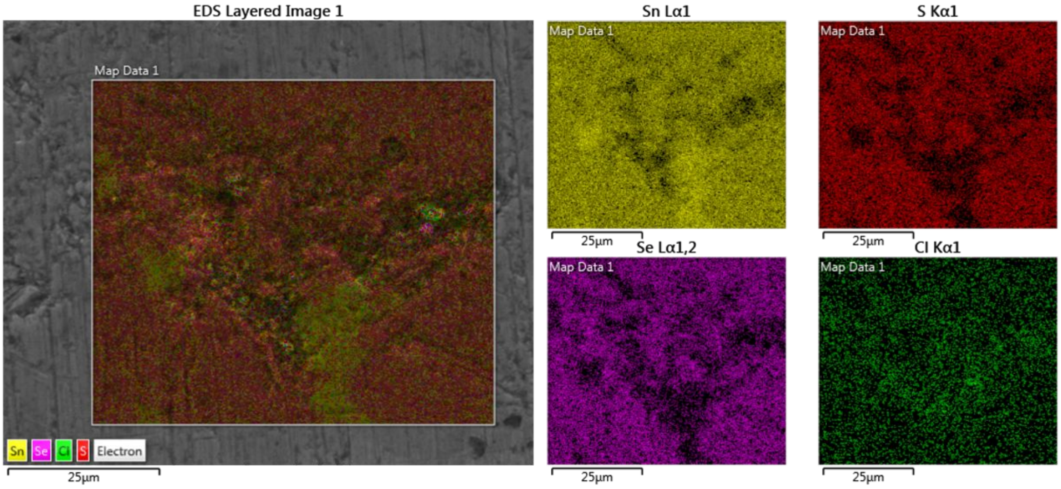

Fig. S9. SEM–EDS elemental maps of Sn (yellow), Se (red), S (magenta), and Cl (green) for $SnS_{0.475}Se_{0.45}$+0.03$SnCl_2$.

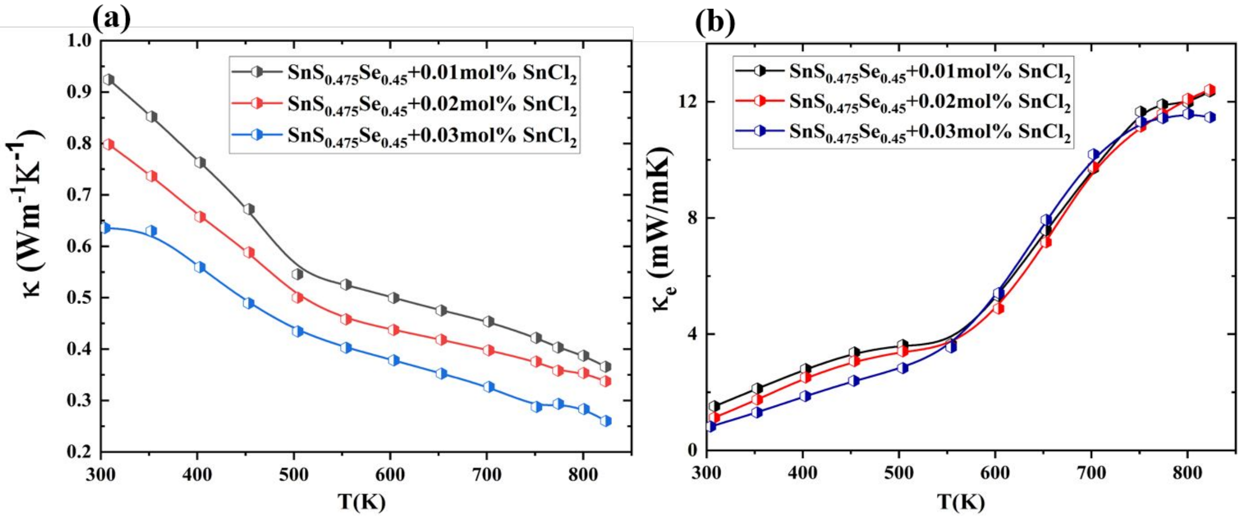

Fig. S10. (a&b) Total and electronic thermal conductivity of the $SnS_{0.905-x}Se_xCl_{0.02}$ (x=0.25, 0.35, 0.45).

Table S1. Lattice constant values of $SnS_{0.905-x}Se_xCl_{0.02}$ (x=0.25, 0.35, 0.45) and $SnS_{0.475}Se_{0.45}$+xmol%$SnCl_2$ samples (x = 0.01, 0.02, 0.03) samples at 300 K.

**Sample**	**a (Å)**	**b (Å)**	**c (Å)**
$SnS_{0.95}$	11.1870	3.9841	4.3244
$SnS_{0.925}$	11.1919	3.9841	4.3291
$SnS_{0.905}Cl_{0.02}$	11.1900	3.9830	4.3283
$SnS_{0.655}Se_{0.25}Cl_{0.02}$	11.1941	3.9856	4.3254
$SnS_{0.555}Se_{0.35}Cl_{0.02}$	11.2813	4.0295	4.3632
$SnS_{0.455}Se_{0.45}Cl_{0.02}$	11.3217	4.0484	4.3815
$SnS_{0.475}Se_{0.45}+0.01SnCl_2$	11.3542	4.0670	4.3907
$SnS_{0.475}Se_{0.45}+0.02SnCl_2$	11.3561	4.0682	4.3910
$SnS_{0.475}Se_{0.45}+0.03SnCl_2$	11.3558	4.0670	4.3915

Table S2. Electrical properties of $SnS_{0.905-x}Se_xCl_{0.02}$ (x=0.25, 0.35, 0.45) samples at 308 K.

**Sample**	**Electrical Resistivity (Ω.m)**	**Seebeck Coefficien t(μV/K)**	**Carrier density(cm^{-3})**	**Mobilit y ($cm^2v^{-1}s^{-1}$)**	**Activation energy (E_a) eV**	**Carrier type**
$SnS_{0.95}$ (380 K)	177.016	-878.45	-9.05×10^{14}	0.31	0.62	n
$SnS_{0.925}$ (380 K)	27.240	-789.13	-6.77×10^{15}	0.34	0.43	n
$SnS_{0.905}Cl_{0.02}$	0.034	-187.31	-2.66×10^{18}	0.68	0.13	n
$SnS_{0.655}Se_{0.25}Cl_{0.02}$	0.017	-228.74	-3.82×10^{18}	0.93	0.10	n
$SnS_{0.555}Se_{0.35}Cl_{0.02}$	0.008	-251.08	-9.24×10^{18}	0.99	0.07	n
$SnS_{0.455}Se_{0.45}Cl_{0.02}$	0.004	-175.25	-1.48×10^{19}	0.85	0.06	n
$SnS_{0.475}Se_{0.45}+0.01SnCl_2$	0.003	-185.01	-6.61×10^{18}	2.74	0.05	n
$SnS_{0.475}Se_{0.45}+0.02SnCl_2$	0.004	-217.95	-5.41×10^{18}	2.64	0.06	n
$SnS_{0.475}Se_{0.45}+0.03SnCl_2$	0.005	-236.23	-3.79×10^{18}	2.33	0.07	n
